# Local Magnetoelectric Effect in La-Doped BiFeO_3_ Multiferroic Thin Films Revealed by Magnetic-Field-Assisted Scanning Probe Microscopy

**DOI:** 10.1186/s11671-016-1534-2

**Published:** 2016-06-30

**Authors:** Dan-Feng Pan, Ming-Xiu Zhou, Zeng-Xing Lu, Hao Zhang, Jun-Ming Liu, Guang-Hou Wang, Jian-Guo Wan

**Affiliations:** National Laboratory of Solid State Microstructures and Department of Physics, Nanjing University, Nanjing, 210093 China; Collaborative Innovation Center of Advanced Microstructures, Nanjing University, Nanjing, 210093 China; Department of Physics and Astronomy, University of Kentucky, Lexington, Kentucky, 40506-0055 USA

**Keywords:** La-doped BiFeO_3_ thin film, Multiferroicity, Magnetoelectric coupling, Scanning probe microscopy, Local conductivity

## Abstract

Multiferroic La-doped BiFeO_3_ thin films have been prepared by a sol-gel plus spin-coating process, and the local magnetoelectric coupling effect has been investigated by the magnetic-field-assisted scanning probe microscopy connected with a ferroelectric analyzer. The local ferroelectric polarization response to external magnetic fields is observed and a so-called optimized magnetic field of ~40 Oe is obtained, at which the ferroelectric polarization reaches the maximum. Moreover, we carry out the magnetic-field-dependent surface conductivity measurements and illustrate the origin of local magnetoresistance in the La-doped BiFeO_3_ thin films, which is closely related to the local ferroelectric polarization response to external magnetic fields. This work not only provides a useful technique to characterize the local magnetoelectric coupling for a wide range of multiferroic materials but also is significant for deeply understanding the local multiferroic behaviors in the BiFeO_3_-based systems.

## Background

Multiferroic materials refer to a class of materials which possess the intrinsic coupling of two or more ferroic orders (e.g., ferroelectricity, ferroelasticity, and ferromagnetism) [1, 2]. Among them, magnetoelectric materials exhibiting both ferroelectric and ferromagnetic characteristics have received much attention due to their great potential in multifunctional applications such as spintronics, memory, and sensors [3–5]. The magnetoelectric coupling between two order parameters makes it possible to manipulate the polarization states by external magnetic fields [6] or vice versa [7, 8]. In single-phase multiferroic materials reported by now, BiFeO_3_ (BFO, with a perovskite ABO_3_ structure) [9] is an ideal candidate which shows unambiguous room-temperature magnetoelectric coupling effect with a Curie temperature of 1103 K and a Néel temperature of 643 K.

In spite of such favorable features, the practical applications of BFO are still limited for several reasons such as formation of secondary phase during preparation, weak magnetoelectric coupling coefficients, and high leakage current [10]. Fortunately, these drawbacks can be overcome by the epitaxial constraints or chemical doping. Suitable ionic doping in the A or B sites of BFO thin films has been widely accepted to improve their magnetic, electric, and magnetoelectric properties [11–14]. Introduction of rare earth ions, particularly La-doped BFO, has been demonstrated to exhibit favorable results [15]. However, the local characterization of magnetoelectric coupling in such multiferroic thin films is still challenging and majority of previous works focus on the macroscopic magnetoelectric effect, while little attention has been paid to its microstructural evolution under either electric or magnetic fields [16, 17]. Besides, it is more important to characterize the local magnetoelectric coupling at nanoscale for practical applications such as high-density data-storage [18]. The recently developed scanning probe microscope (SPM) techniques provide us an efficient way to study the local magnetoelectric coupling in multiferroic materials, accompanied with remarkable advantages in their non-invasive character, versatility, and high spatial resolution [19, 20].

In this work, by means of the magnetic-field-assisted scanning probe microscopy connected with an external ferroelectric tester, we systematically investigate the local magnetoelectric coupling behaviors in the multiferroic La-doped BiFeO_3_ polycrystalline thin films. The local ferroelectric polarization as well as the surface current distribution under external magnetic fields is observed. Based on the analysis about the change of average surface conductivity under various magnetic fields, we clarify the local magnetoelectric coupling process and the origin of local magnetoresistance in the La-doped BiFeO_3_ thin films, both of which are closely related to the local ferroelectric polarization response to external magnetic fields.

## Methods

### Sample Preparation

The Bi_0.9_La_0.1_FeO_3_ (BLFO) thin films were prepared on the commercial Pt/Ti/SiO_2_/Si (100) substrates by a typical sol-gel process and spin-coating technique. In detail, bismuth nitrate (Bi(NO_3_)_3_ · 5H_2_O), ferric nitrate (Fe(NO_3_)_3_ · 9H_2_O), and lanthanum nitrate (La(NO_3_)_3_ · 6H_2_O) were chosen as starting materials. Acetic acid and 2-methoxyethanol (1:4, volume ratio) were the solvent. The BLFO precursor solution was obtained by dissolving the nitrates into 2-methoxyethanol. Considering the loss of bismuth in the preparation process, 5 % excess Bi(NO_3_)_3_ · 5H_2_O was added. After being ultrasonic cleaned sequentially in deionized water, acetone, and ethanol, the Pt substrate was dried by N_2_. Then the precursor BLFO solution was spin-coated onto the substrate at 500 rpm for 8 s and 4000 rpm for 30 s in a glove box full of N_2_. Before coating the next layer, the film was dried at 200 °C for 5 min at a heating plate. The thickness of BLFO film was controlled by repeating times of the spin-coating procedure. Finally, the film was annealed at 550 °C for 10 min under oxygen atmosphere. The detailed preparation can also be found elsewhere [21].

### Characterization and Measurements

The structural characteristic of the as-prepared BLFO film was carried out by the X-ray diffraction (XRD) analysis on a D/MAX-RA diffractometer with Cu Kα radiation. A scanning electron microscopy (SEM, ULTRA 55-44-08) was used to examine the cross-sectional morphology. The magnetization vs. magnetic field (M-H) hysteresis loop was measured using a superconducting quantum interference device (SQUID, Quantum Design). We used an atomic force microscopy (AFM) (NT-MDT Inc.) to characterize the surface topography, and the surface current distribution mapping was obtained by using the attached conductive-atomic force microscopy (c-AFM) with a Cr/Pt-coated tip. The tip which acted as a mobile top electrode was grounded, and the bias voltage was applied to the bottom electrode. The ferroelectric domain structures were revealed by piezoresponse force microscopy (PFM), and the external magnetic fields were generated by the Helmholtz coils from NT-MDT Inc. For local electrical measurements, tiny circular electrodes with the diameter of 20 μm were made by a conventional photolithography method [22]. The local polarization vs. voltage (P-V) hysteresis loops were obtained by a standard ferroelectric analyzer (Precision Multiferroic, Radiant Inc.) connected to the AFM controller using a Signal Access Module (NT-MDT Inc.). The local I-V curve was measured by the single-point c-AFM mode. For comparison, macroscopic I-V measurements were also performed. Forty-nanometer Au electrodes were deposited onto the film using ion-sputtering technique through a shadow mask with the diameter of 0.2 mm, and the I-V curve was recorded by a Keithley 6517B electrometer. Without special descriptions, all the measurements were performed at room temperature and under ambient condition.

## Results and Discussion

### Basic Characterizations of the BLFO Thin Films

**Fig. 1:**
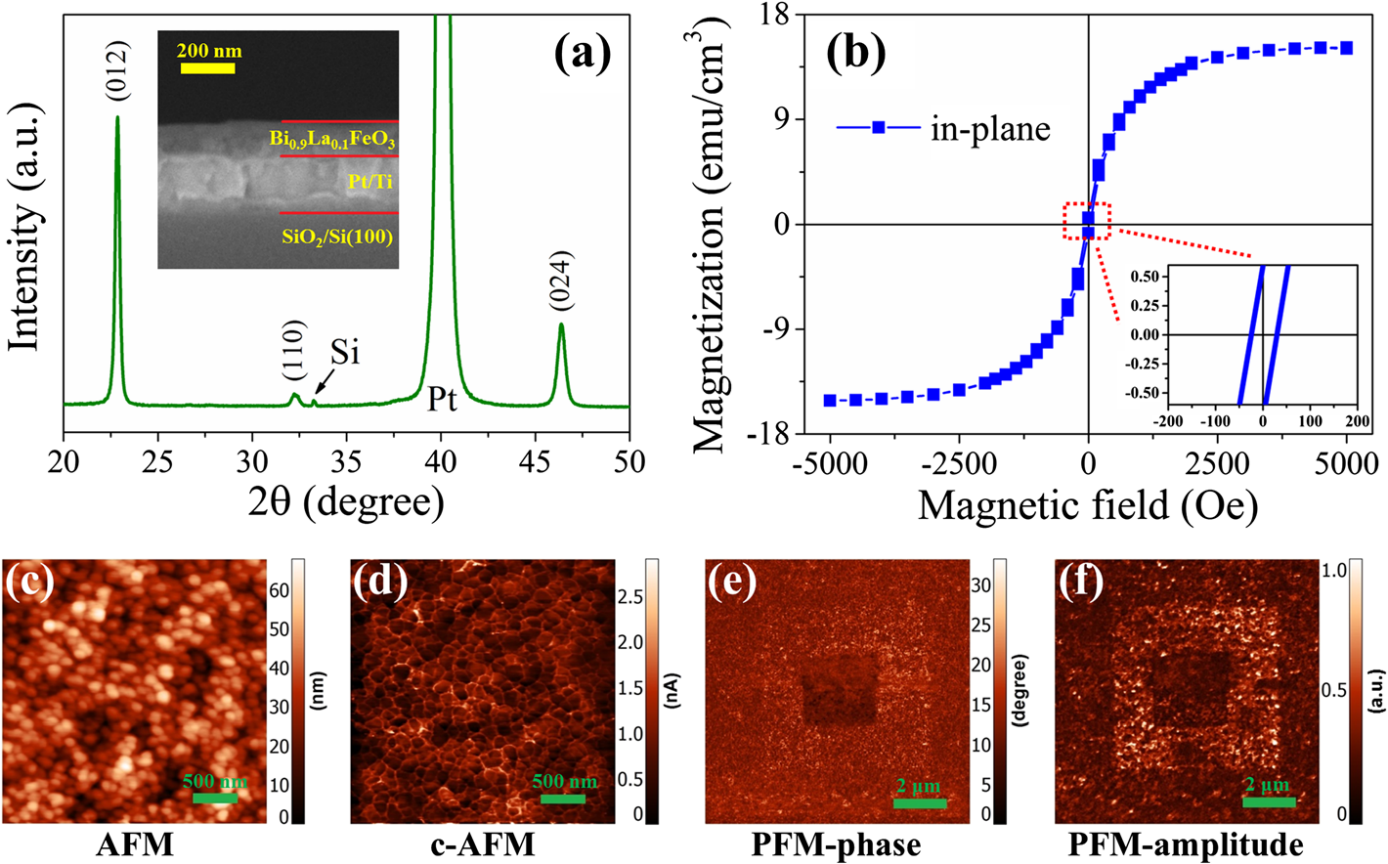
**a** XRD pattern of the BLFO film. The *inset* shows the cross-sectional SEM image. **b** M-H hysteresis loop of the BLFO film. The *inset* gives the expanded area of the red dotted circle. **c** Surface AFM image of the BLFO film with the area of 3 × 3 μm^2^. **d** The corresponding c-AFM image at a bias voltage of 6 V. **e**, **f** The typical 10 × 10 μm^2^ out-of-plane PFM phase and amplitude images, respectively

Figure 1a presents the XRD pattern of the as-prepared BLFO thin film. All peaks are identified for the rhombohedral perovskite structure of the BLFO phase, and no other impurity or intermediate phases are observed. The inset of Fig. 1a gives the cross-sectional SEM image of the BLFO film deposited on the Pt/Ti/SiO_2_/Si (100) substrate, which shows distinguishable interfaces between each layer and determines the thickness of the film to be ~120 nm. To confirm the multiferroic property of the BLFO film, we firstly measured the room-temperature M-H hysteresis loops, as shown in Fig. 1b. The film exhibits a weak ferromagnetism with the saturation magnetization of ~15 emu/cm^3^. From the expanded picture in the inset of Fig. 1b, we obtained the coercive magnetic field to be ~20 Oe. The weak ferromagnetism in the present La-doped BFO film could be ascribed to the suppression on the intrinsic cycloidal spin structures of BFO, which is caused by the substrate-induced strain and the lattice distortion of La-doping [23].

Figure 1c presents the typical surface AFM topographic image of the chosen 3 × 3 μm^2^ BLFO film. It can be observed that the as-prepared BLFO film is well-crystallized and the average grain size is about 100 nm. Besides, the film is rather dense and the average surface roughness is ~30 nm. Figure 1d gives the corresponding c-AFM mapping at a bias voltage of 6 V with the same area. It is easily concluded that the grain boundary is more conductive than the grain interior, which is consistent with our previous work [24]. The local electrical conduction is attributed to the oxygen vacancies accumulated at the grain boundaries of polycrystalline BLFO film. We then performed the PFM measurements to test the local polarization response to external electric fields. The chosen 6 × 6 μm^2^ area was firstly written with +6 V bias voltage using a biased conductive tip; then, a central smaller square of 3 × 3 μm^2^ was reversely poled with −6 V bias voltage in the same way; finally, in the outer 10 × 10 μm^2^ area, both the PFM-phase and PFM-amplitude images were recorded under zero bias voltage, as depicted in Fig. 1e, f. Clearly, the BFLO film shows a switchable ferroelectric response to external electric fields, and the ferroelectric domains are altered to the opposite direction upon the application of reversed voltage. The above M-H and switchable PFM experimental results confirm the existence of both ferroelectricity and ferromagnetism in the present BLFO thin films.

### Local Ferroelectric Polarization Behaviors Under External Magnetic Fields

**Fig. 2:**
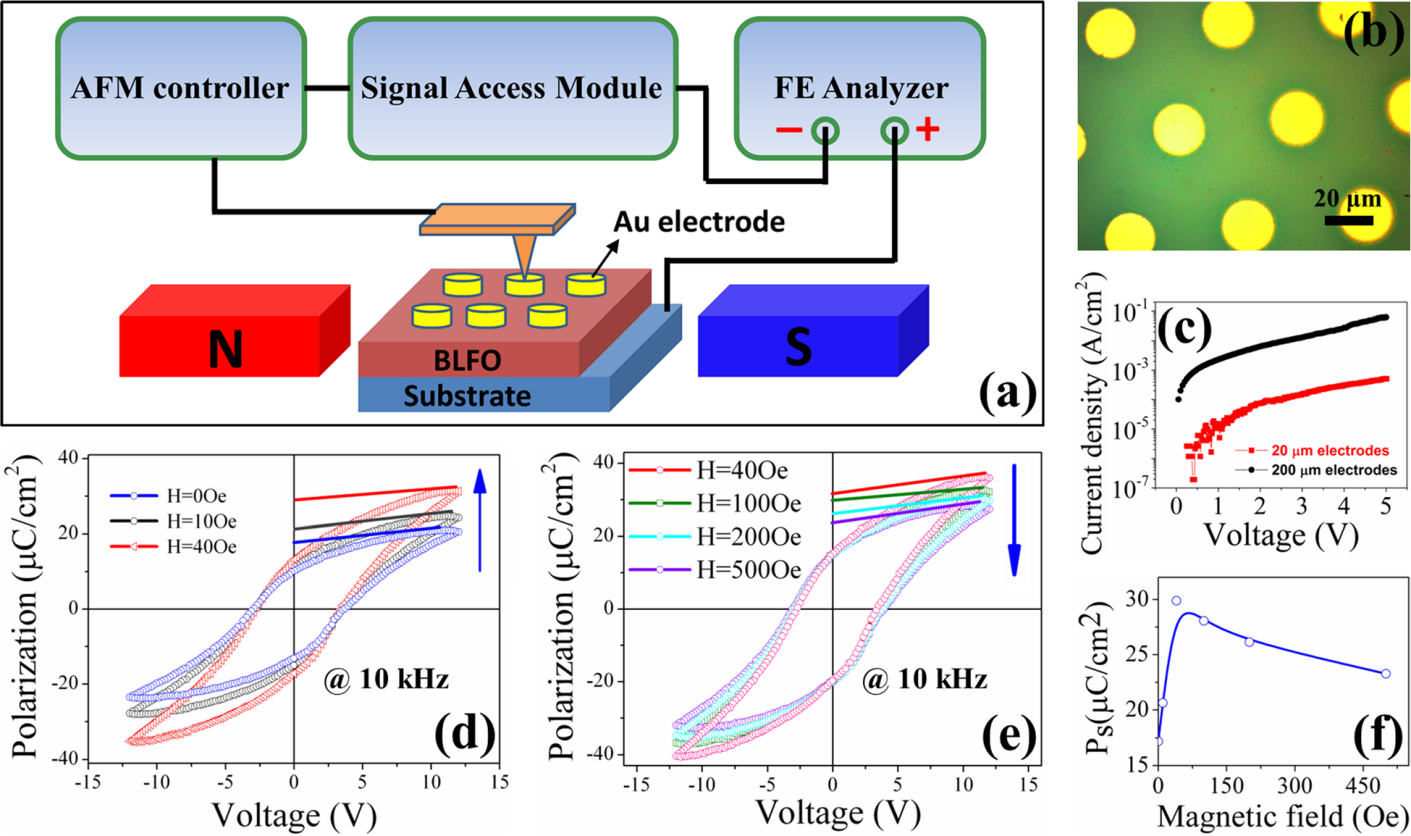
**a** The schematic diagram of local P-V hysteresis loop measurements under magnetic fields. **b** Optical micrograph of the tiny electrodes with the diameter of 20 μm by a photolithography method. **c** Comparison of the local leakage current density vs. voltage curve with 20 and 200 μm electrodes. **d**, **e** The local ferroelectric P-V hysteresis loops under various magnetic fields ranging from 0 to 40 Oe and 40 to 500 Oe, respectively. **f** The saturation polarization (*P*_s_) values as a function of external magnetic fields derived from **d** and **e**

Measuring the ferroelectric polarization under external magnetic fields is an effective way to characterize the magnetoelectric coupling in multiferroic materials [25]. However, in the multiferroic thin films coated by top electrodes with the size of several hundred micrometers, the true change of ferroelectric polarization manipulated by external magnetic fields cannot be quantitatively figured out due to the existence of high leakage current. In order to eliminate the influence of leakage current on the polarization current to the greatest extent, we should use tiny electrodes to reduce the extra-conductive path induced by various defects in the preparation process of BLFO film as much as possible. In this work, we developed an effective characterization technique combining the magnetic-field-assisted scanning probe microscopy with external ferroelectric analyzer to study the local magnetoelectric coupling in BLFO thin film. Figure 2a illustrates the schematic diagram of the measurement setup. The AFM controller is connected to external ferroelectric analyzer by a Signal Access Module, and the AFM tip is used to probe the tiny electrodes. Figure 2b presents the optical micrograph of the tiny electrodes made by a typical photolithography method. The diameter of the circle electrodes is ~20 μm. Figure 2c plots the local leakage current with tiny electrodes using the single-point c-AFM mode. For compassion, we also measured the leakage current for the BLFO thin film coated by 200-μm-diameter electrodes. Typically, under an applied 5 V voltage, the local leakage current density is only 10^−3^ A/cm^2^, nearly two orders of magnitude lower than the macroscopic leakage tests. Thus, a confirmable change of the local ferroelectric polarization under external magnetic fields can be expected.

Subsequently, we carried out the local P-V hysteresis loop measurements under various magnetic fields, as shown in Fig. 2d, e. All the tests were performed intentionally at 10 kHz to further exclude the influence of leakage current. With no magnetic field applied, a well-defined ferroelectric polarization hysteresis loop was obtained with a saturation polarization (*P*_s_) of ~18 μC/cm^2^. Upon the application of external magnetic fields, the P-V hysteresis loops emerge an obvious change with the external magnetic fields, indicative of evident magnetoelectric coupling in the BLFO thin film. The *P*_s_ value increases sharply when the external magnetic field is applied from 0 to 40 Oe. However, when the external magnetic field is above 40 Oe, the *P*_s_ value starts to decrease. Figure 2f gives the *P*_s_ values derived from Fig. 2d, e as a function of external magnetic fields.

In general, the ferroelectric and magnetic characteristics in a BFO system are coming from two independent parts, i.e., the ferroelectric polarization is originated from the displacement of Bi^3+^ ions caused by the active 6 s^2^ loan pair while the magnetic property mainly comes from the Fe^3+^ ions and related cycloidal spin structures [26]. The vectors of the ferroelectric polarization and antiferromagnetic spin are strongly coupled in a typical BFO unit. The Dzyaloshinskii-Moriya interaction can lead to the canting of the magnetic moments and the appearance of macroscopic magnetization, which causes the polar distortion in BFO [27]. In the BFO-based films with suppressed spiral spin structures, the orientation of spin vectors is coupled with ferroelectric polarization vectors through the ferroelastic strain. When a magnetic field is applied to the BFO-based films, the recanting of the magnetic moments happens, which facilitates the antiferromagnetic domain switching and reduces the activation energy for the switching of the electrical polarization domains [28]. This further gives rise to a change in the ferroelectric domain switching behaviors, causing the variation of ferroelectric polarization under external magnetic fields. In addition, during the magnetoelectric coupling measurements, the leakage current can also affect the observed polarization value under external magnetic fields. In fact, since the background leakage current is inevitably superimposed on the polarization current, the observed polarization value under external magnetic fields is slightly larger than the actual values.

### Local Electric Conduction Characteristics Under External Magnetic Fields

**Fig. 3:**
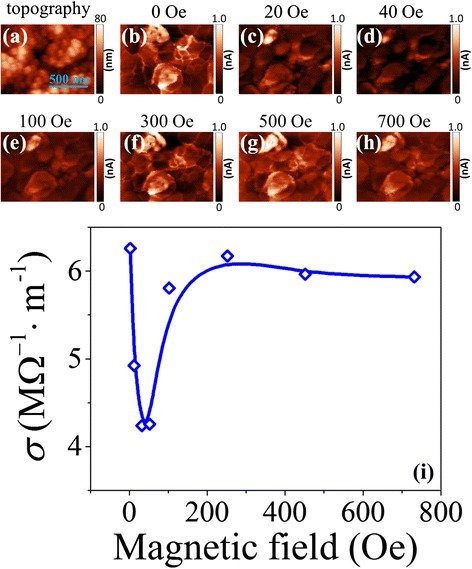
**a**–**h** The surface topography and corresponding c-AFM images at a 6 V bias voltage under different magnetic fields of the chosen 1.5 × 1 μm^2^ area. **i** Average surface electrical conductivity as a function of external magnetic fields calculated from **b**–**h**

According to previous investigations, the local electrical conduction behavior in the BLFO thin film is mainly contributed to the conduction of grain interiors and grain boundaries, both of which are closely related to the distribution of oxygen vacancies in the film [24]. Considering the variation of ferroelectric domain switching behaviors can definitely influence the redistribution of oxygen vacancies in the BLFO thin film, we suggest that the local electrical conduction behaviors should vary with external magnetic fields. The magnetic-field-assisted c-AFM measurements were carried out at a given bias voltage of 6 V under different magnetic fields, as shown in Fig. 3(a–h). Both the surface morphology and surface current mappings were recorded simultaneously by the contact mode. With no magnetic field applied (Fig. 3b), the grain interiors and boundaries show different extents of conductivity, in which the grain boundary conductivity parts originate from the accumulation of oxygen vacancies while the grain interior conductivity parts are related to the ferroelectric domains [29]. However, the grain boundary conductivity dominates the whole conductivity in the measurement area. When the external magnetic field is applied, evident changes in the brightness and contrast of the current mappings can be observed. With increasing the magnetic field from 0 to 700 Oe, the same area exhibits different conductive characteristics as a series of c-AFM configurations (Fig. 3b–h). From Fig. 3b–h, we calculated the average electrical conductivity of the whole measuring area under different magnetic fields and depicted the corresponding values as a function of magnetic fields in Fig. 3i. At ~40 Oe, the conductivity value is decreased to the minimum and gets back to a high level when the magnetic field increases again, which is consistent with the ferroelectric polarization behaviors under magnetic fields (Fig. 2f).

We consider that such a local electric conduction behavior under external magnetic fields should be closely related to the ferroelectric polarization response to magnetic fields. Before applying a magnetic field, the oxygen vacancies are randomly distributed in the polycrystalline BLFO thin films. These oxygen vacancies are easily trapped or accumulated at the grain boundaries and domain walls [30]. The presence of oxygen vacancies is a main reason for the large leakage current [31]. When applying a magnetic field to the BLFO thin film, due to the existence of local mangetoelectric coupling in BLFO, the switching behaviors of microscopic ferroelectric domains will change, accompanied with the variation of local electric fields. For compensating the discontinuity of local polarization, the accumulated oxygen vacancies will redistribute due to the switching of ferroelectric domains, consequently leading to the change in local conductive behaviors.

## Conclusions

In summary, the local magnetoelectric coupling behaviors were systematically investigated in the multiferroic BLFO polycrystalline thin films. The local ferroelectric polarization response to magnetic fields confirmed the robust existence of local magnetoelectric coupling, and a so-called optimized magnetic field of ~40 Oe was obtained, at which it exhibited the strongest magnetoelectric coupling effect. The further magnetic-field-dependent local conductivity tests showed a local magnetoresistance effect in the film, which could be attributed to the redistribution of bound oxygen vacancies dominated by the polarization-dependent local ferroelectric domain switching under external magnetic fields. This work provides a useful technique to characterize the local magnetoelectric coupling in multiferroic materials and is also beneficial for studying the intrinsic coupling of multiple ferroic orders in BiFeO_3_-based systems.
